# Non-Contact Respiratory Monitoring Using an RGB Camera for Real-World Applications

**DOI:** 10.3390/s21155126

**Published:** 2021-07-29

**Authors:** Chiara Romano, Emiliano Schena, Sergio Silvestri, Carlo Massaroni

**Affiliations:** Unit of Measurements and Biomedical Instrumentation, Università Campus Bio-Medico di Roma, Via Alvaro del Portillo, 00128 Rome, Italy; c.romano@unicampus.it (C.R.); e.schena@unicampus.it (E.S.); s.silvestri@unicampus.it (S.S.)

**Keywords:** breathing, contactless monitoring systems, respiratory monitoring, RGB cameras

## Abstract

Respiratory monitoring is receiving growing interest in different fields of use, ranging from healthcare to occupational settings. Only recently, non-contact measuring systems have been developed to measure the respiratory rate ($f_{R}$) over time, even in unconstrained environments. Promising methods rely on the analysis of video-frames features recorded from cameras. In this work, a low-cost and unobtrusive measuring system for respiratory pattern monitoring based on the analysis of RGB images recorded from a consumer-grade camera is proposed. The system allows (i) the automatized tracking of the chest movements caused by breathing, (ii) the extraction of the breathing signal from images with methods based on optical flow (FO) and RGB analysis, (iii) the elimination of breathing-unrelated events from the signal, (iv) the identification of possible apneas and, (v) the calculation of $f_{R}$ value every second. Unlike most of the work in the literature, the performances of the system have been tested in an unstructured environment considering user-camera distance and user posture as influencing factors. A total of 24 healthy volunteers were enrolled for the validation tests. Better performances were obtained when the users were in sitting position. FO method outperforms in all conditions. In the $f_{R}$ range 6 to 60 breaths/min (bpm), the FO allows measuring $f_{R}$ values with bias of −0.03 ± 1.38 bpm and −0.02 ± 1.92 bpm when compared to a reference wearable system with the user at 2 and 0.5 m from the camera, respectively.

## 1. Introduction

The monitoring of vital signs, such as the respiratory rate, heart rate, body temperature, and blood pressure, is essential to assess the general health status [1]. Among others, respiratory rate ($f_{R}$) and its potential as a predictor of sickness state have been often neglected [2,3]. However, respiratory monitoring is receiving growing interest in different fields of use, ranging from healthcare to occupational settings and sport. In clinical settings, $f_{R}$ monitoring moves from intensive care to inpatient wards, being an indicator of severe systemic imbalances [2]. For instance, it has been shown that $f_{R}$ of 25–29 breaths per minute (bpm) is associated with a mortality rate of 21% [4]. Additionally, value of $f_{R}$ strongly correlates with early detection of high-risk conditions, such as obstructive sleep apnea (OSA) which is a sleep breathing disorder characterized by partial or complete obstruction of the upper airway during sleep. OSA is estimated to affect between 10% and 17% of adult men and 3% and 9% of adult women and studies suggest that at least 80% of individuals with OSA are undiagnosed [5,6]. In occupational settings, due to its sensitivity to cognitive load, stress and other factors, $f_{R}$ is used to monitor workers by improving health and safety [7,8]. Finally, in the sporting field in order to optimize training and improve the performance of athletes, especially in precision sports [9]. Methods requiring direct contact with the subject are typically used to record breathing related events and to calculate the $f_{R}$ from inhaled and exhaled flow variations, airflow temperature changes, chest wall circumference changes, cardiac modulation of the breathing [10]. Hallmarks of these techniques reduce their acceptability, they require sensors which can be expensive, may cause skin irritation and discomfort especially in long-term monitoring or during sleep, and may influence the physiological parameters during monitoring [11]. For these reasons, there has been an increased interest in developing non-contact methods. They can be used as remarkable solutions in different application fields, especially in the scenarios where unobtrusive methods are required (e.g., hospital waiting rooms, tele-monitoring, neonatal intensive care units). Some examples in clinical settings include Doppler radar [12], depth sensors [13], laser vibrometry [14], and RGB cameras [13,15,16]. Additionally, different non-contact techniques including thermal cameras, video cameras, and radar sensors can be used in the automotive environment, or even for monitoring cognitive load and emotional stress in computer workers [17].

Among all non-contact methods, those based on optical sensors integrated into commercial video cameras introduce several advantages, such as low cost, the possibility of being used by a non-expert, and ease of use [16].

There are different studies in which video cameras have been tested to monitor $f_{R}$ that differ mainly in the method of signal extraction from sequential images, in the posture of the subject, and in the location of the region of interest (ROI) from which the signal is extracted. For example, ref. [18] uses variation in intensity of RGB image pixels within a selected ROI at the pit of the neck to select a respiratory signal, ref. [19] tracks head movements by means the averaging of the red channel. In addition, ref. [20] tracks the deviation of selected feature points near the upper torso and head over the time and [21,22] use the optical flow method (hereinafter, FO). All the above-mentioned methods give good results in acquiring the respiratory pattern, but they have some limitations. The algorithms presented in [18,19] are highly dependent on ambient light variations not related to respiration and they require operator intervention in choosing the ROI. In the method proposed in [20] if there is no compensation by the tracking algorithm of small head motions, the estimated respiration will be inaccurate. Moreover, although [21,22] use FO methods, as in our work, there are some differences. In fact, ref. [21] uses Lucas Kanade’s local FO over the entire image, unlike our work in which the Horn Schunck dense FO is used within a selected region of interest at the chest level. Using the FO over the entire image requires a much longer computational time and also the system is not robust to breathing unrelated movements as any movement of the subject is detected and computed. Hence, there would then be a need for additional post-processing of the data to eliminate these unwanted movements. Ref. [22] uses the phase of the calculated FO on healthy subjects while seating from a lateral perspective. However, this method necessarily requires the subject to be placed in a lateral position. It also evaluates motion from the phase of the FO, unlike our study, in which only the modulus of the FO is considered. In addition, all these methods have only been tested in a structured environment, with a limited number of subjects.

In literature, there are only a few studies aiming at investigating performances with different user postures and distance from the video camera [23] and that present a method for detection and elimination of artifacts in the signal. Moreover, although there are plenty of studies that focus on detecting respiration through cameras, literature lacks studies aiming at comparing different techniques for the respiratory signal extraction. Finally, most of the works neglected the continuous estimation of $f_{R}$ values which are needed for monitoring purposes. All these points still limit the use of these non-contact systems in real-life applications.

Within this context, the method that we propose tries to overcome the above mentioned limitations through a non-contact and unobtrusive measuring system for respiratory monitoring based on the analysis of RGB video-frames recorded with a mobile device for respiratory monitoring. The method includes an automatic selection of the ROI and a method for the recognition and elimination of movements not related to the breathing activity. Moreover, compared with the literature in which a narrower range of $f_{R}$ is investigated, we demonstrated that $f_{R}$ and apnea phase can be assessed by the system over a wide range of $f_{R}$ (10 to 42 bpm) including quiet breathing (12 bpm ≤ $f_{R}$ ≤ 20 bpm) and its alterations, as well as tachypnea ($f_{R}$ > 20 bpm) and bradypnea ($f_{R}$ < 12 bpm). We have investigated two different techniques to retrieve respiratory signals from the video-frames (the first based on the pixel intensity changes and the second based on FO) at different distances between the user and the camera (i.e., 0.5, 1.5, 2.0 m) and different user’s postures (i.e., standing, sitting, supine) in an indoor unstructured environment (i.e., varied clothing both in terms of pattern and color, variable ambient light, non-homogeneous background, other people were allowed to pass behind the subject during the test) simulating like those of daily living. Finally, we performed a signal analysis capable of estimating the $f_{R}$ values with an update time of 1 s, unlike most studies that only estimate the average $f_{R}$ in the post-processing analysis.

## 2. Materials and Methods

A video captured with a CCD camera can be considered as a sequence of RGB frames (polychrome images). Each frame in RGB space is an image matrix consisting of primary elements called pixels to which an intensity level in the form of a numerical value expressed in bits is associated. In commercial RGB cameras, as in this work, 8 bits per channel are used (24-bit for RGB space). Indeed, images in RGB space are characterized by the property that each color can be represented using the superposition of three values (i.e., one for each channel), which encode the intensities of red (R), green (G), and blue (B) contributing to the specific color. Therefore, each RGB frame can be seen as a two-dimensional (*x*,*y*) distribution of intensity I(x,y), where I(x,y) = $I_{R}\left(x,y\right)$ + $I_{G}\left(x,y\right)$ + $I_{B}\left(x,y\right)$. I(x,y) depends on two main components:

- A component proportional to the amount of direct light incident on the scene, called the illumination component i(x,y);
- A component proportional to the amount of light reflected by objects in the scene, called the reflectance r(x,y).

These two components combined give origin to the intensity distribution of the scene, as in Equation (1).

(1) I(x,y)=i(x,y)⁢r(x,y)

where 0<I(x,y)<∞,0<i(x,y)<∞,0<r(x,y)<1 [24].

### 2.1. Breathing-Related Chest Wall Motion Extraction from Video-Frames

Respiratory activity causes cyclical movements of the chest wall characterized by an expansion of the rib cage during inhalation, resulting in an upward movement of the thorax and a relaxation of the same during exhalation, resulting in a downward movement of the thorax. This cyclic movement generates consecutive changes in reflected light intensity that can be used to indirectly monitor respiratory activity through a CCD camera. In this work, two techniques (i.e., pixel intensity changes and FO) have been used to post-process data to extract the user’s respiratory pattern and, then, the $f_{R}$ values, as described in the following subsection.

### 2.2. Proposed Algorithms

**Figure 1:**
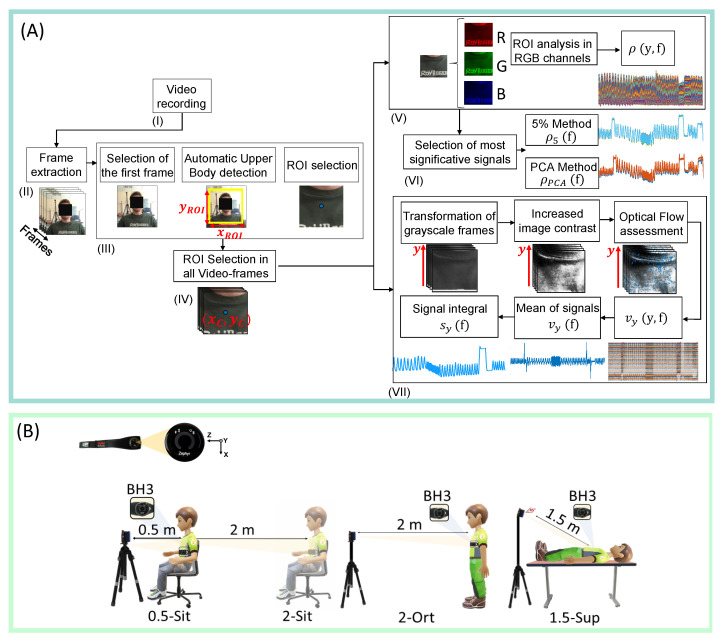
(**A**). Flowchart presenting the steps carried out to extract the respiratory pattern from image sequences. (**B**). Experimental set-up (0.5-Sit: user-camera distance of 0.5 m, sitting position; 2-Sit: user-camera distance of 2 m, sitting position; 2-Sta: user-camera distance of 2 m, standing position; 1.5-Sup: user-camera distance of 1.5 m, supine position). BH3: Bioharness, used as reference instrument.

Once the video is acquired from the RGB camera (Figure 1(AI)), a region of interest (ROI) that contains breathing-related information must be identified in each frame. In order to do this, after the video-frames are extracted (Figure 1(AII)), an automatic algorithm is applied to detect and select the upper body (UB) in the first frame (Figure 1(AIII)), as the body area between the face and shoulders. To accomplish this task, Viola–Jones object detection algorithm is applied [25].

Starting from the identified UB, of size $x_{UB}\times y_{UB}$, the proposed algorithm selects a point located in $\left(x_{C},y_{C}\right)=\left(\frac{x_{UB}}{2},0\right)$ which determines the central point of the ROI from which the respiratory signal will be extracted. From this point, the rectangular ROI is extracted with dimensions $x_{ROI}$ × $y_{ROI}$, as in Equations (2) and (3):

(2) $$x_{ROI}=k\;\,\left[x_{C}-10\%\,Video_{width},x_{C}+10\%\,Video_{width}\right]$$

(3) $$y_{ROI}=k\;\,\left[y_{C}-5\%\,Video_{height},y_{C}+5\%\,Video_{height}\right]$$

where *k* is a coefficient that is inversely proportional to the distance of the subject from the camera and used to tackle the size variability of the ROI with the distance from the camera. Then, the ROIs in all video-frames are extracted (Figure 1(AIV)). All the above-mentioned steps are needed to apply two post-processing methods used to retrieve the respiratory pattern: the variation of light intensity and the FO method which are briefly described in the following two subsections.

#### 2.2.1. Pixel Intensity Changes

By recording a video of the chest wall region with a camera, the red, green, and blue (RGB) channels collect a mixture of the reflected signal together. So that, at each frame, three different intensity signals are recorded (i.e., one for each channel), as in Figure 1(AV).

Since the respiratory signal is pseudoperiodic, it is possible to associate the respiratory pattern with the periodic intensity of the pixels variations over time [18].

Applying this method, an intensity signal is obtained over time for each pixel in the RGB channels. Therefore, at each frame *f*, the intensity components of each channel I(x,y,c,f) are obtained, where *c* is the color channel (i.e., red (R), green (G), and blue (B)). The proposed algorithm sums the intensity components obtained in the three channels and then averages them for each line *y* of the ROI, according to the following equation:

(4) $$\rho \left(y,f\right)=\frac{1}{x_{ROI}}\,\sum_{x=1}^{x_{ROI}}\,\left(\sum_{c=R,G,B}\,I\left(x,y,c,f\right)\right),\;\;y\in \left[1,y_{ROI}\right]$$

In this way, the intensity component ρ(y,f) is obtained for each row of the ROI per each frame *f* (Figure 1(AV)). To retrieve the respiratory pattern, an additional step is required to reduce the dimensionality of ρ(y,f). Among methods that can be used to select the most informative signals among all the ρ(y,f), we used the PCA and the 5% method which have been demonstrated promising in similar applications [26,27]. The PCA selects the signals that constitute 95% of the variance explained, whereas the 5% method selects 5% of the signals with the highest standard deviation, as in Figure 1(AVI).

#### 2.2.2. Optical Flow

When the FO method is applied to video-frames, a prior transformation from images in RGB space to grey-scale images is required. Among others, the Lukas–Kanade [28], the Farneback [29] and the Horn and Shunk (HS) [30] are the most used algorithms to extract the FO from the frames.

Focusing on HS, the algorithm formulation assumes that pixels conserve their intensity along their trajectory. According to the assumption of constant brightness, the intensity of a pixel I(x,y,f) at the frame *f* will remain stable for short time and small movements. For a single frame step df, the following equation is valid:

(5) I(x,y,f)=I(x+dx,y+dy,f+df)

dx and dy denote the displacements in *x* and *y* direction. Assuming that the pixel displacement is sufficiently small, the following equation is obtained:

(6) $$\frac{\partial I}{\partial x}\,v_{x}+\frac{\partial I}{\partial y}\,v_{y}+\frac{\partial I}{\partial f}=0$$

where $v_{x}$ and $v_{y}$ are the pixel velocity components along the *x*-axes and *y*-axes of the FO of I(x,y,f) that are to be determined. This equation with two unknown variables cannot be solved [30]. To overcome this issue the HS algorithm computes the displacement between two consecutive images by tracking the image features on a pixel-by-pixel basis. In this way, a velocity vector for each pixel in the image is obtained.

In this paper, only the velocity component along the *y*-axis—$v_{y}\left(y,f\right)$—was chosen as it was assumed to be the one most related to the movements of the rib cage caused by breathing. After extracting the ROI from all video-frames, a grey-scale image transformation is performed and the image contrast is increased by saturating the bottom 1% and the top 1% of all pixel values using imadjust (a MATLAB function), to improve the FO performance. Finally, the HS optical flow was applied to all video-frames. Assuming that within the ROI the direction of the y-velocity vectors agree and that the modulus value is similar for almost all vectors, all values within the ROI were averaged to obtain a single value for each frame. This results in a single average velocity vector ($v_{y}$). Finally, the velocity vector was integrated in order to obtain rib cage linear displacement related to the respiratory activity ($s_{y}$), as in Figure 1(AVII).

## 3. Experimental Setup and Protocol

In total, 24 healthy volunteers (i.e., 15 males, 9 females, mean age 26 ± 4 years old, mean height of 170 ± 7 cm, mean body mass 70 ± 13 kg) were enrolled in this study to investigate the performance of the proposed measuring system with the proposed algorithms. Per each volunteer, trials were carried out at different postures (i.e., sitting, standing, and supine) and user-camera distances (0.5, 1.5, 2.0 m), as shortly summarized in Table 1 where 0.5-Sit is related to user-camera distance of 0.5 m, sitting position; 2-Sit to user-camera distance of 2 m, sitting position; 2-Sta to user-camera distance of 2 m, standing position and 1.5-Sup to user-camera distance of 1.5 m, supine position.

All the tests were carried out in compliance with the Ethical Approvals (ST-UCBM 27/18 OSS) and, prior to the tests, all the participants provided their informed consent. All the trials were carried out following COVID-19 restrictions (i.e., face mask use and social distancing).

**Figure 2:**
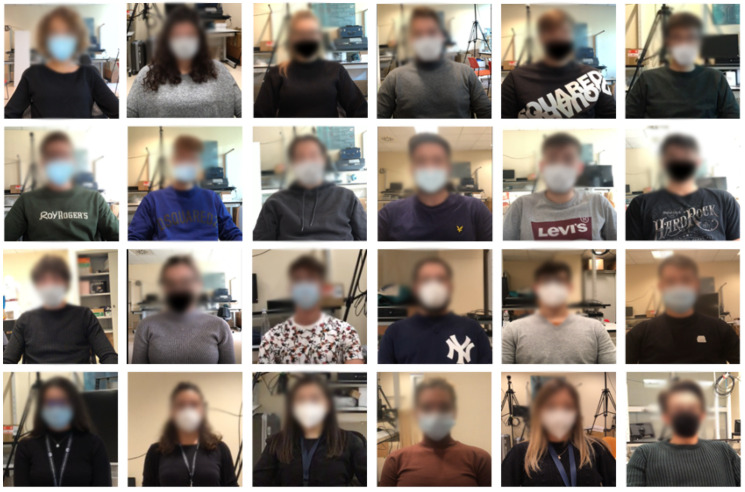
A picture of all the subjects just before the test is shown in the figure in order to show the variety of clothing worn.

To capture the video, the built-in smartphone RGB camera (iPhone 6s, Apple Inc., Cupertino, CA, USA) was used. The camera was configured to acquire 30 frames per second (fps) with a high definition resolution (i.e., 720p, *x* = 1280 px, *y* = 720 px). All experiments were carried out indoor and with a stable amount of light delivered by neon lights. No restrictions have been placed on the clothing of the subjects, as shown in Figure 2.

A multi-parameter wearable device, the Zephyr BioModule BioHarness 3 by Medtronic (hereinafter, BH3), was used to record the reference respiratory signal contextually to the video recording. This system consists of a thoracic belt and an electronic module and acquires the breathing pattern of the user by sensing the volumetric changes in the thorax by the means of a strain gauge [31]. The reference breathing signal was sampled at 25 Hz.

**Figure 3:**
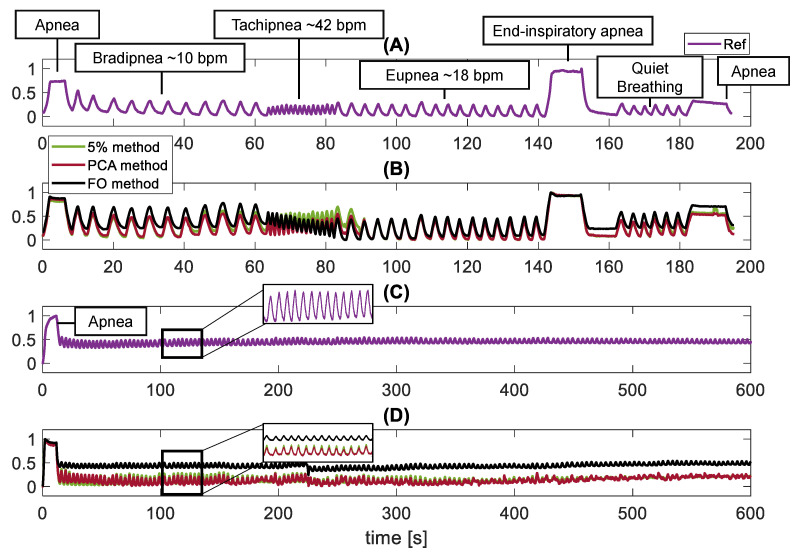
The 4 trends show the protocol that volunteers were asked to follow. In (**A**,**B**) the protocol consists of apnea for ∼5 s, one minute of bradypnea ($f_{R}$ ∼ 10 bpm), 20 s of tachypnea ($f_{R}$ ∼ 42 bpm), one minute of eupnea ($f_{R}$ ∼ 12 bpm), ∼10 s of end-inspiratory apnea, ∼10 s of end-expiratory apnea, quiet breathing for ∼10 s and a final apnea. Whereas trends (**C**,**D**) show the protocol that volunteers were asked to follow in the 1.5-sup trial that includes an initial apnea of approximately 5 s and quiet breathing for approximately 600 s. In detail: (**A**) Breathing pattern recorded by the BH3 during a seated trial (0.5-Sit). The different respiratory stages are briefly indicated. (**B**) The same breathing pattern extracted from the three methods based on video processing. (**C**) Example of a breathing pattern recorded by the BH3 during a supine trial (1.5-Sup). (**D**) The same breathing pattern extracted from the three methods based on video processing.

In the first three trials (see Figure 1B) each participant was asked to stand in front of the camera (i.e., sitting on a chair or standing) at a distance of 0.5 m and 2 m from the camera. These distances were chosen because, in line with literature, they are those most investigated in the occupational and clinical field. Then, the experimenter set the camera so that the chest area of the subject was framed (see an example in Figure 1(AII)). Each subject was guided to perform the steps indicated by a graphical user interface running on a laptop placed on a desk behind the camera and visible to the volunteer. The graphical interface was developed to standardize the protocol, which included: warm-up breathing (not considered in the analysis), apnea for ∼5 s, one minute of bradypnea ($f_{R}$ ∼ 10 bpm), 20 s of tachypnea ($f_{R}$ ∼ 42 bpm), one minute of eupnea ($f_{R}$ ∼ 12 bpm), ∼10 s of end-inspiratory apnea, ∼10 s of end-expiratory apnea, quiet breathing for ∼10 s and a final apnea, as in Figure 3A. The subject’s breathing pattern was guided using an animation timing the inhalation and exhalation phases, as well as apnea stages. An example of the BH3 signal recorded during a standing trial is shown in Figure 3A, while the signals extracted by the video with the 5%, PCA and FO methods are shown in Figure 3B. In the last trial (see Figure 1B), 5 volunteers were enrolled (3 males and 2 females, mean age 26 ± 2 years old, mean height of 165 ± 15 cm, mean body mass 68 ± 13 kg) and called to breathe spontaneously for ∼600 s after a short initial apnea used to synchronize instruments. An example of the BH3 signal recorded during a 1.5-Sup trial is shown in Figure 3C, while the signals extracted by the video with the 5%, PCA and FO methods are shown in Figure 3D.

## 4. Data Analysis and Results

The collected videos were post-processed in MATLAB environment to extract the breathing patterns with the proposed algorithms. Per each trial, we retrieved $\rho_{5}\left(f\right)$, $\rho_{PCA}\left(f\right)$ by processing the video-frames with the pixel intensity changes method and $s_{y}\left(f\right)$ with the FO method. Therefore, the $\rho_{5}\left(f\right)$, $\rho_{PCA}\left(f\right)$ and $s_{y}\left(f\right)$ were synchronized with the reference signal (hereafter, Ref) by using the first and last apneas as common events (see Figure 3A). A third-order Butterworth low-pass filter with a cut-off frequency of 2 Hz was applied to all the signals [18]. This choice preserves the constant signal related to apnea and, at the same time, deletes the high frequencies related to noise, guaranteeing a wide range of detectable $f_{R}$ values (i.e., up to 120 bpm).

In this paper, we compared the $f_{R}$ extracted from the $\rho_{5}\left(f\right)$, $\rho_{PCA}\left(f\right)$ and $s_{y}\left(f\right)$ signals against those retrieved from the post-processing of Ref signal over-time, at the different user’ postures and user-camera distances. Moreover, we investigated the performance of the proposed measuring system in detecting apneas.

### 4.1. Respiratory Frequency Monitoring over Time

To monitor the $f_{R}$ over time in real-world applications, motion artefacts caused by breathing-unrelated events must be identified and removed. Additionally, apneas that can occur during the continuous monitoring especially in clinical scenarios, must be identified.

#### 4.1.1. Motion Artefacts Removal

Before starting monitoring the $f_{R}$ values over time, any artefacts not related to respiratory activity from the signal must be removed.

A typical artefact is characterized by a sudden increase in signal amplitude and a sudden change in breathing pattern rhythm compared to the previous and following time instants. At this aim, the following tasks were carried out:

1. The derivative of the signals extracted from the video (i.e., $\rho_{5}\left(f\right)$, $\rho_{PCA}\left(f\right)$ and $s_{y}\left(f\right)$) are calculated;
2. The amplitude of the derivative of the signals are compared against a threshold (i.e., th) defined as three times the standard deviation of the signal derivative;
3. The signal with derivative outside the interval ±th is considered as an artefact.

**Figure 4:**
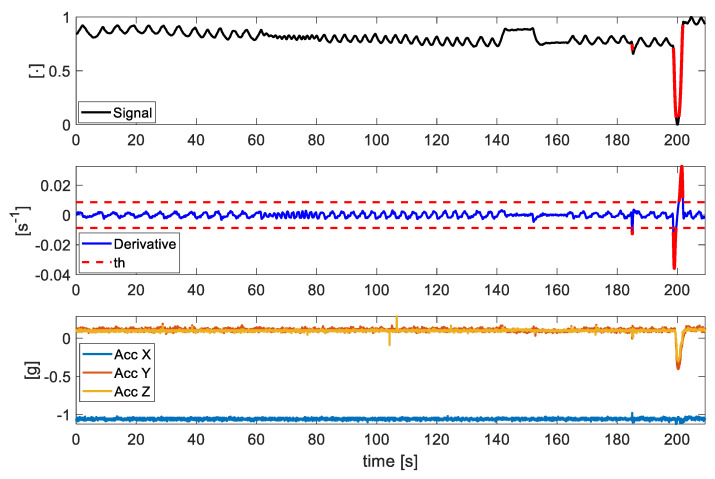
In the first subplot, an exemplary signal extracted by the FO method from a video; in the second subplot the derivative of this signal and the th represented as red dashed lines; in the third subplot the *X*, *Y*, and *Z* axis of the accelerometer inside the BH3 (see Figure 1B) used as reference of the breathing-unrelated events. In red, the artefacts identified on the signal and derivative.

As an example, the first subplot in Figure 4 shows a signal extracted using the FO method in which the portions identified as artefacts are automatically highlighted in red, according to the threshold method defined above and showed in the second subplot. This is supported by the third subplot in which the X, Y, and Z axis of the accelerometer inside the BH3 are reported. The acceleration magnitude shows large variations in correspondence with the event recognized. In all the trials that present motion artefacts, the events were correctly identified and removed from the $\rho_{5}\left(f\right)$, $\rho_{PCA}\left(f\right)$ and $s_{y}\left(f\right)$ signals (data are not shown).

#### 4.1.2. Apnea Detection

After the motion artefacts removal, the apnea stages were identified in all the signals. A typical apnea stage is characterized by a reduced signal variation. To automatically identify the apneas in all the signals we performed the following steps:

- The derivatives of the signals extracted from the video (i.e., $\rho_{5}\left(f\right)$, $\rho_{PCA}\left(f\right)$ and $s_{y}\left(f\right)$) are calculated;
- The standard deviation of the derivatives are calculated over 30 s windows, with 29 s overlapping and used as a threshold (i.e., sd);
- Whether the derivative of the signal is less than the previously calculated standard deviation for at least 10 s (in accordance with guidelines in [32]), the signal at those points is identified as apnea.

**Figure 5:**
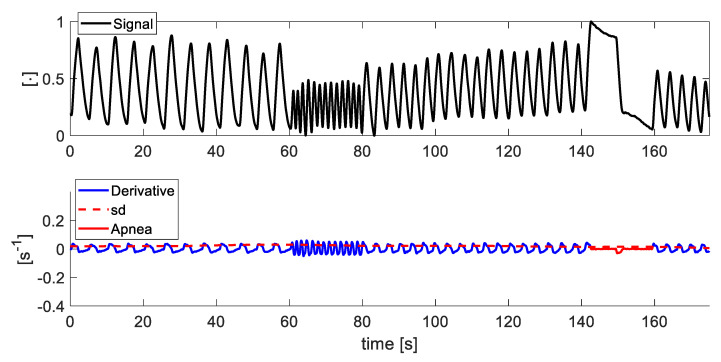
In the first subplot, a signal obtained by the FO method from a video without the portions of the signal identified as artefacts; in the second subplot the derivative of the signal and the sd represented as red dashed lines. In red, the apneas identified on the derivative.

As an example, the first subplot in Figure 5 shows a signal extracted using the FO method from which the portions of the signal identified as artefacts have been removed and then the portions identified as apnea are automatically highlighted in red, according to the threshold method defined above and showed in the second subplot. In order to evaluate the performance of the apnea detection method, only the tests in which each subject was asked to hold breath (end-expiratory and end-inspiratory apneas), see Figure 3, for about 10 s are taken into account. The apneas detected by this method at the portions in which the subject was asked to hold breath in the protocol were identified as correctly detected apneas. Finally, signals in which the apnea recognition method identifies as apnea a portion of the signal in which the subject did not hold breath were considered as false positives. Table 2 reports the detected, not detected, and false positive apneas considering the signals recorded by all the volunteers. In all the cases (posture and distances) the FO method better perform when compared to other methods with a 96% of the apneas correctly detected in the case of seated volunteers at 0.5 m and 2 m and 75% of cases with the subject in standing position. Then, 5% and the PCA methods presents the worst performances in all the trials. For all the methods, the standing position presented the higher undetected apneas (up to 75% when PCA was used) which can be explained by the postural sway of the body that does not occur in seated conditions [33].

#### 4.1.3. Respiratory Frequency Calculation

After this stage, the first 120 s of each trial were used for the analysis. To compute the $f_{R}$ values, we applied a 30 s sliding window with 29 s overlapping on each signal to obtain $f_{R}$ values with an update time of 1 s. In each window the following steps were carried out per each signal:

- Removing the mean from the signal (i.e., detrending);
- Normalization between 0 and 1, so that even lower amplitude peaks were detectable within the window;
- Identification of all the local maxima on the signal by using a MATLAB findpeaks (min amplitude to identify the peak set on 20%), in accordance with [34];
- Calculation of the *n* breathing periods ($T_{n}$) obtained as the time elapsed between the consecutive maxima (*n* is the number of identified breaths), and of the average breathing period ($T_{w}$);
- Calculation of the window $f_{R}$ value as the $\frac{60}{T_{w}}$.

**Figure 6:**
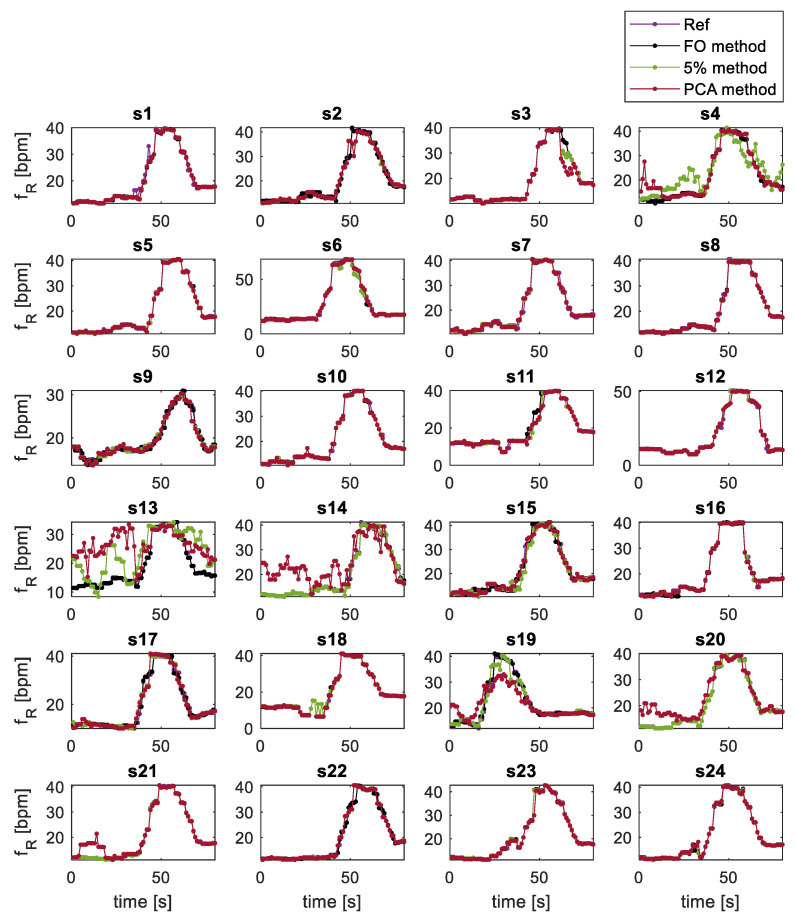
Trend of $f_{R}$ expressed in bpm extracted every second with the optical flow method (in black), 5% method (in green), PCA method (in red) and with the reference (in purple) on all subjects for the first 90 s of the 2-sit test.

**Figure 7:**
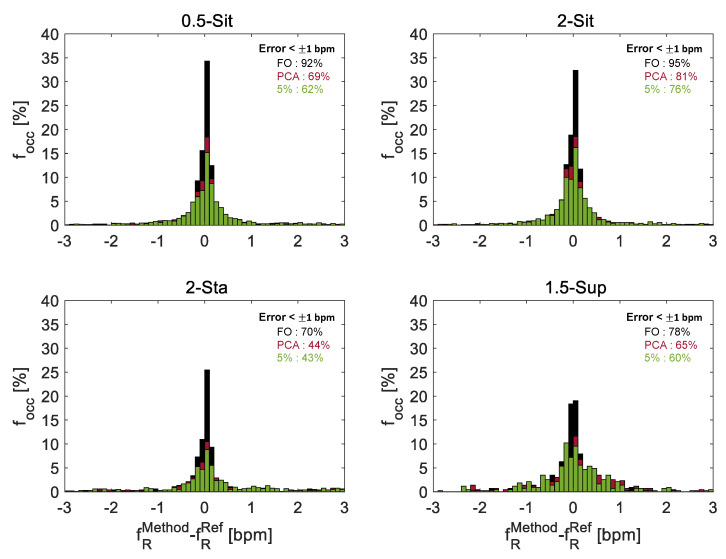
Distributions of the error ($f_{R}^{Method}-f_{R}^{Ref}$) per each trial and for each technique (in black FO, in red PCA and in green 5%). On the y-axis the percentage occurrence frequency. In the box, the percentage of the measurements with errors below ±1 bpm.

**Figure 8:**
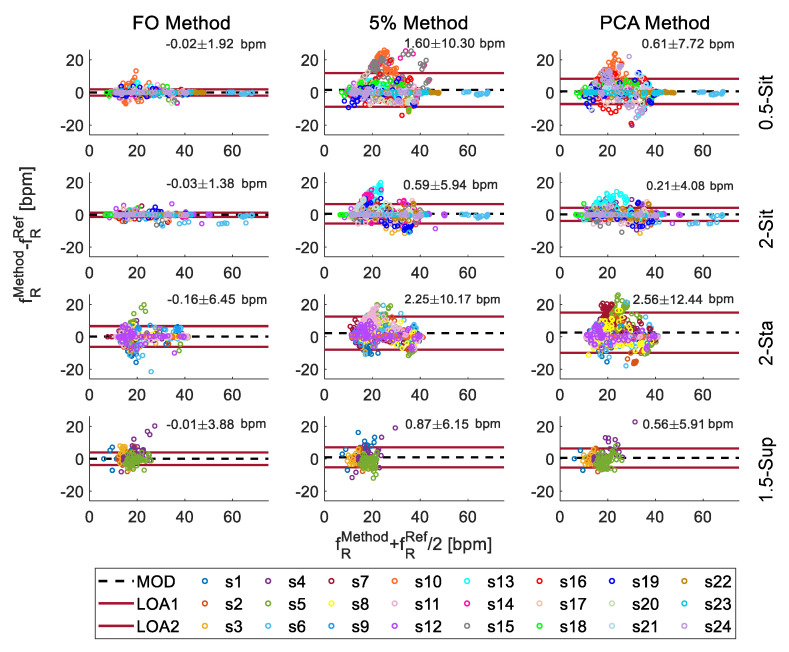
Bland–Altman analysis using the $f_{R}$ values obtained by the video signal collected for each subject (shown in different colours) posture and for each technique of extracting movement from images. The black dashed line represents the mean of difference (MOD); the two upper and lower lines represent the confidence interval given by the mean ± the 95% of the standard deviation of the points (LOAs). MOD can be interpreted as an indicator of the accuracy of the system and its value represents the systematic error of the proposed method to the reference. The smaller the variation of the mean within the confidence interval, the more likely it is that the two methods can be considered interchangeable.

An example in which the extracted second-by-second respiratory rate values for all subjects with all extraction techniques is shown in Figure 6. The trends of $f_{R}$ extracted with all methods show a first part in which the signal is settled around 10 bpm, followed by an increasing respiratory rate of about 42 bpm that finally falls to about 18 bpm according to the protocol. From the Figure 6 for all subjects, except for subjects 4, 13, 14, 19, there is almost complete overlap of the 3 methods with the reference. On the other hand, in the above-mentioned subjects there is a not very good overlap of the 5% method and PCA method with the reference, while the FO method is almost completely overlapped. These errors are quantified with the histograms shown in Figure 7 and with the Bland Altman plots showed in Figure 8.

The $f_{R}$ values obtained each second from $\rho_{5}\left(f\right)$, $\rho_{PCA}\left(f\right)$ and $s_{y}\left(f\right)$ were compared to those obtained from Ref. Figure 7 reports the distribution of the differences between $f_{R}$ values computed from each method (i.e., 5%, PCA, FO) and those calculated by the reference (i.e., Ref), by considering all the volunteers in the different trials. In all the postures, the FO method showed lower errors than the other two methods: the best results are achieved in 2-Sit trial using the FO method, of $f_{R}$ values presented 95% errors below ±1 bpm when compared to reference values, while in the 5% method and the PCA method only the 76% and 81% of $f_{R}$ values were in that range, respectively.

To compare the $f_{R}$ values obtained from each method every 1 s against the Ref values we used the Bland–Altman analysis. We used the $f_{R}$ values retrieved from all the volunteers to investigate the mean of differences (MOD) and limits of agreements (LOAs) at the different user-camera distances and postures [35].

In all the trials, at all the user-camera distances and postures, the FO presented the best performances (see first column in Figure 8) compared to the other methods. $f_{R}$ values extracted from video-frames when the user was seated (i.e., 0.5-Sit and 2-Sit trials) showed the lowest bias (expressed as MOD ± LOAs) with all 3 methods compared to other trials, with bias of −0.03 ± 1.38 bpm with FO method, 0.59 ± 5.94 bpm with 5% method and 0.21 ± 4.08 bpm with PCA method. Considering all the trials, Bland–Altman analysis shows lower MOD values when FO method is used (max −0.16 bpm) and greater overlap with the reference than the other two techniques. Differently from PCA and 5% methods, FO slightly underestimates the values, showing a negative MOD in all tests. LOAs were used to investigate the error dispersion. FO method performs better having LOAs of ±1.38 bpm in 2-Sit trial. However, even in the worst case the LOAs are not far off, being ±1.92 bpm, much lower values than 5% method and PCA method which in the worst case present LOAs of 10.17 bpm and 12.44 bpm, respectively.

## 5. Discussion

Respiratory monitoring is receiving growing interest in different fields of use, ranging from healthcare to occupational settings. Especially, $f_{R}$ is used to monitor workers to improve health and safety in a working environment or also as an indicator of severe systemic imbalances in clinical settings [2]. Although in the last years several studies investigated different techniques based on the analysis of videos for retrieving respiratory parameters from images, literature lacks studies aiming at comparing signal extraction techniques, and only few studies aimed at assessing the influence on signals related to subject postures and distance from the video camera [36]. Lastly, even if the breathing-unrelated events are well known to affect the signals in both wearable and contactless techniques, rarely video-based techniques includes motion artefact removal algorithms [16].

In this paper, we presented a non-contact measurement system for the respiratory pattern and $f_{R}$ monitoring based on the analysis of video images collected by optical sensors integrated into commercial cameras. We have investigated the performance in several scenarios resembling those of real-world scenarios characterized by possible different user-camera distances, user postures, presence of motion artefacts, and apneas. We developed and tested algorithms working on video-frames to: (i) select in an automatic and optimized way the ROI from which the signal is extracted, allowing, also, long-term monitoring with a computational burden suitable for a commercial laptop; (ii) automatically eliminate non-breathing related events; (iii) identify apneas; (iv) estimate $f_{R}$ values with an update time of 1s. To investigate the performance of the proposed methods, experiments were carried out under challenging conditions: (i) unstructured environment; (ii) varied clothing both in terms of pattern and color; (iii) different postures of the subject (sitting, standing, supine) and different distances from the camera (0.5–2 m). Moreover, volunteers were called to breathe in a wide range of $f_{R}$ including quiet breathing and its alterations (i.e., bradypnea and tachypnea).

Considering all the trials, the FO method allowed detecting 96% of the apneas in the signals up to 2 m while user seated, while performances drop down to 38% in standing position (62% undetected) at 2 m of distance from the camera. These results are in line with [37], in which machine learning techniques are applied to respiratory signals to recognize apnea. Regarding the $f_{R}$ monitoring over-time, all the methods presented better performances when the user was seated, probably due to the absence of the postural sway of the body that occur in standing position. FO method performed better than others when compared against values provided by the BH3, showing error below ±1 bpm in the 95% of cases. Bland–Altman analysis evidenced lower bias when FO method is used in all the cases. FO method allow monitoring $f_{R}$ with bias of −0.02 ± 1.92 bpm and −0.03 ± 1.38 bpm in the seated postures which is comparable to those obtained in similar conditions with wearable devices [38] (MOD ± LOAs = 0.01 ± 2.39 bpm) and better than those obtained with other contactless techniques based on video [22] (MOD ± LOAs = 0.08 ± 1.48 bpm), radar [39] (MOD ± LOAs = −1.21 ± 6.99 bpm), and thermal cameras [40] (MOD ± LOAs = −0.30 ± 2.69 bpm). Errors found with the user in the supine position (bias up to −0.01 ± 3.88 bpm) are comparable to those of [41] (MOD ± LOAs = 0.36 ± 2.50 bpm) in which a combination of near-infrared and thermal imaging techniques are used.

## 6. Conclusions

The results obtained in this study are promising in the context of monitoring the $f_{R}$ over-time in different postures and without specific boundary conditions (e.g., specific garment pattern or specific ambient light conditions). However, it is necessary to clarify that the main limitation is that all the $f_{R}$ calculation were carried out with a post-processing and also that the FO computational complexity has been found to be higher compared to PCA and 5% methods. These considerations must be considered for further real-time monitoring applications. Further tests will be helpful to test the proposed system in real-world scenarios. We are already carrying out data acquisition in office. Moreover, because of the good performances in the breathing pattern monitoring, additional tests will be helpful to the study of respiratory asymmetries using multiple ROIs in different parts of the abdomen and thorax which is quite complicated with wearable systems.

## Figures and Tables

**Table 1:** Experimental Protocol: distances, postures, durations, and enrolled volunteers in each trial.

Trial	Distance	Posture	Trial Duration	Number of Subjects
0.5-Sit	0.5 m	Sitting	∼4 min	24
2-Sit	2 m	Sitting	∼4 min	24
2-Sta	2 m	Standing	∼4 min	24
1.5-Sup	1.5 m	Supine	∼10 min	5

**Table 2:** Apneas detection results.

Method	Trial	Detected	Not Detected	False Positive	#Apnea
FO method	0.5-Sit	96%	4%	26%	48
	2-Sit	96%	4%	39%	48
	2-Sta	38%	62%	12%	48
5% method	0.5-Sit	75%	25%	25%	48
	2-Sit	79%	21%	29%	48
	2-Sta	33%	67%	8%	48
PCA method	0.5-Sit	71%	29%	29%	48
	2-Sit	75%	25%	38%	48
	2-Sta	25%	75%	8%	48

## Data Availability

The data presented in this study are available on request from the corresponding author. The data are not publicly available due to privacy reason.

## Abbreviations

The following abbreviations are used in this manuscript:

$f_{R}$	Respiratory rate
bpm	breaths/min
OSA	Obstructive sleep apnea
ROI	Region of interest
px	Pixel
x${}_{C}$	Location on x axis of the ROI’s central point
y${}_{C}$	Location on y axis of the ROI’s central point
FO	Optical flow
PCA	Principal component analysis
ICA	Independent component analysis
UB	Upper body
HS	Horn and Shunk
BH3	Bioharness
th	Threshold
f${}_{occ}$	Percentage occurrence frequency
MOD	Mean of differences
LOA	Limit of agreement
sd	Standard deviation
